# Supplementary material for: Arsenic and mercury tolerant rhizobacteria that can improve phytoremediation of heavy metal contaminated soils
Source: PeerJ. 2023 Jan 12;11:e14697. doi: 10.7717/peerj.14697 (PMC9840862; doi:10.7717/peerj.14697)
Supplement: Supplemental Information 1 [file peerj-11-14697-s001.docx]

Tabla 3. Tolerance to the heavy metals arsenic and mercury of isolates growing in the presence of heavy metal salts

| Metallic salt/ concentration**†** | TL 1 | TL 2 | TL 7 | TL 23 | TL 35 | TL 36 | TL 49 | TL 52 | TL 65 | TL 80 | TL 97 |
| --- | --- | --- | --- | --- | --- | --- | --- | --- | --- | --- | --- |
| HAsNa_2_O_4_ |  |  |  |  |  |  |  |  |  |  |  |
| 200 mg/kg | **+** | **+** | **+** | **+** | **+** | **+** | **+** | **+** | **+** | **+** | **+** |
| 400 mg/kg | **+** | **+** | **+** | **+** | **+** | **+** | **+** | **+** | **+** | **+** | **+** |
| 600 mg/kg | **+** | **+** | **+** | **+** | **+** | **+** | **+** | **+** | **+** | **+** | **+** |
| 800 mg/kg | **+** | **+** | **+** | **+** | **+** | **+** | **+** | **+** | **+** | **+** | **+** |
| 1000 mg/kg | **+** | **+** | **+** | **+** | **+** | **+** | **+** | **+** | **+** | **+** | **+** |
| ‍ |  |  |  |  |  |  |  |  |  |  |  |
| AsNaO_2_ |  |  |  |  |  |  |  |  |  |  |  |
| 200 mg/kg | **+** | **+** | **+** | **+** | **+** | **+** | **+** | **+** | **+** | **+** | **+** |
| 400 mg/kg | **+** | **+** | **+** | **+** | **+** | **+** | **+** | **+** | **+** | **+** | **-** |
| 600 mg/kg | **+** | **+** | **+** | **+** | **+** | **+** | **+** | **+** | **+** | **-** | **-** |
| 800 mg/kg | **+** | **+** | **+** | **+** | **+** | **+** | **+** | **+** | **+** | **-** | **-** |
| 1000 mg/kg | **+** | **+** | **+** | **+** | **+** | **+** | **+** | **+** | **+** | **-** | **-** |
| ‍ |  |  |  |  |  |  |  |  |  |  |  |
| Cl_2_Hg |  |  |  |  |  |  |  |  |  |  |  |
| 23 mg/kg | **-** | **-** | **-** | **+** | **-** | **+** | **+** | **+** | **-** | **+** | **+** |
| 50 mg/kg | **-** | **-** | **-** | **+** | **-** | **+** | **+** | **+** | **-** | **+** | **+** |
| 75 mg/kg | **-** | **-** | **-** | **-** | **-** | **+** | **+** | **+** | **-** | **+** | **-** |
| 100 mg/kg | **-** | **-** | **-** | **-** | **-** | **-** | **-** | **-** | **-** | **-** | **-** |
| 150 mg/kg | **-** | **-** | **-** | **-** | **-** | **-** | **-** | **-** | **-** | **-** | **-** |

**Notes:** (**+**) Growth of colonies in the presence of As (V), As (III), and Hg (I). (**-**) No growth of colonies in the presence of As (V), As (III), and Hg (I).
