# Supplementary material for: Arsenic and mercury tolerant rhizobacteria that can improve phytoremediation of heavy metal contaminated soils
Source: PeerJ. 2023 Jan 12;11:e14697. doi: 10.7717/peerj.14697 (PMC9840862; doi:10.7717/peerj.14697)
Supplement: Supplemental Information 3 [file peerj-11-14697-s003.docx]

Table 1. Bacterial species isolated from three soil samples from the Tlalpujahua, Michoacán, Mexico mining region identified based on their fatty acid profiles

| Isolate | Source | Species | % Similarity (MIDI) |
| --- | --- | --- | --- |
| TL1 | Sample 1 | *Paucimonas lemoignei* | 0.303 |
| TL2 | Sample 1 | *Pseudomonas alcaligenes* | 0.677 |
| TL3 | Sample 1 | *Citrobacter amalonaticus* | 0.471 |
| TL4 | Sample 1 | *Acinetobacter calcoaceticus* | 0.708 |
| TL6 | Sample 1 | *Pseudomonas alcaligenes* | 0.473 |
| TL7 | Sample 1 | *Acinetobacter calcoaceticus* | 0.512 |
| TL8 | Sample 1 | *Herbaspirillum huttiense* | 0.355 |
| TL11 | Sample 2 | *Stenotrophomonas maltophilia* | 0.278 |
| TL21 | Sample 2 | *Sphingobacterium faecium* | 0.378 |
| TL22 | Sample 2 | *Pedobacter heparinus* | 0.221 |
| TL23 | Sample 2 | *Paracoccus denitrificans* | 0.840 |
| TL24 | Sample 2 | *Rhodobacter sphaeroides* | 0.747 |
| TL30 | Sample 2 | *Paracoccus denitrificans* | 0.901 |
| TL31 | Sample 2 | *Cellulomonas fimi*-GC subgroup A | 0.613 |
| TL33 | Sample 3 | *Brevibacillus centrosporus* | 0.558 |
| TL34 | Sample 3 | *Bacillus alcalophilus* | 0.568 |
| TL35 | Sample 3 | *Brevibacillus choshinensis* | 0.681 |
| TL36 | Sample 3 | *Herbaspirillum huttiense* | 0.496 |
| TL37 | Sample 3 | *Kocuria rosea*-GC subgroup A | 0.768 |
| TL38 | Sample 3 | *Pseudomonas fluorescens* | 0.582 |
| TL39 | Sample 3 | *Bacillus megaterium* | 0.639 |
| TL40 | Sample 3 | *Paenibacillus validus* | 0.432 |
| TL41 | Sample 3 | *Staphylococcus gallinarum*-GC subgroup A | 0.597 |
| TL43 | Sample 3 | *Paenibacillus alvei-*GC subgroup A | 0.454 |
| TL44 | Sample 3 | *Microbacterium barkeri* | 0.819 |
| TL47 | Sample 3 | *Paenibacillus pabuli* | 0.656 |
| TL48 | Sample 3 | *Brevundimonas vesicularis* | 0.771 |
| TL49 | Sample 3 | *Klebsiella oxytoca*-GC subgroup B | 0.528 |
| TL51 | Sample 3 | *Bacillus*-GC group 22 | 0.551 |
| TL52 | Sample 2 | *Rhizobium radiobacter* | 0.811 |
| TL54 | Sample 2 | *Rhodococcus wratislaviensis* | 0.706 |
| TL55 | Sample 2 | *Microbacterium barkeri* | 0.695 |
| TL62 | Sample 2 | *Acinetobacter calcoaceticus* | 0.708 |
| TL64 | Sample 2 | *Nocardia otitidiscaviarum* | 0.477 |
| TL65 | Sample 2 | *Paenibacillus polymyxa* | 0.563 |
| TL67 | Sample 2 | *Pantoea agglomerans* | 0.344 |
| TL68 | Sample 2 | *Bacillus-*GC group 22 | 0.707 |
| TL69 | Sample 2 | *Bacillus-*GC group 22 | 0.587 |
| TL74 | Sample 2 | *Sphingopyxis macrogoltabida* | 0.894 |
| TL76 | Sample 2 | *Bacillus atrophaeus* | 0.492 |
| TL79 | Sample 2 | *Bacillus alcalophilus* | 0.568 |
| TL80 | Sample 3 | *Pseudomonas putida* | 0.161 |
| TL84 | Sample 3 | *Stenotrophomonas maltophilia* | 0.649 |
| TL85 | Sample 3 | *Rhizobium rubi* | 0.645 |
| TL86 | Sample 3 | *Klebsiella oxytoca-*GC subgroup B | 0.401 |
| TL91 | Sample 3 | *Pseudomonas fluorescens* | 0.634 |
| TL93 | Sample 3 | *Herbaspirillum huttiense* | 0.492 |
| TL97 | Sample 3 | *Pseudomonas fluorescens* | 0.907 |
