# Supplementary figures and images for: Arsenic and mercury tolerant rhizobacteria that can improve phytoremediation of heavy metal contaminated soils
Source: PeerJ. 2023 Jan 12;11:e14697. doi: 10.7717/peerj.14697 (PMC9840862; doi:10.7717/peerj.14697)

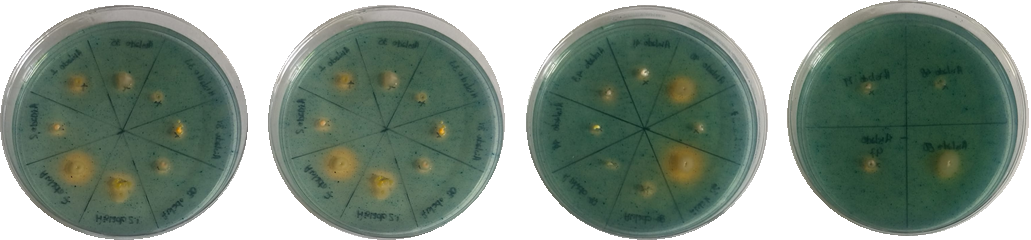

Supplement: Supplemental Information 4 — Evaluation of siderophore production in isolates from soil samples from Tlalpujahua, Michoacán, Mexico, cultivated in chrome azurol sulfonate (CAS) agar medium incubated at 30 °C. The orange rings surrounding the colonies were measured daily. [file peerj-11-14697-s004.png]

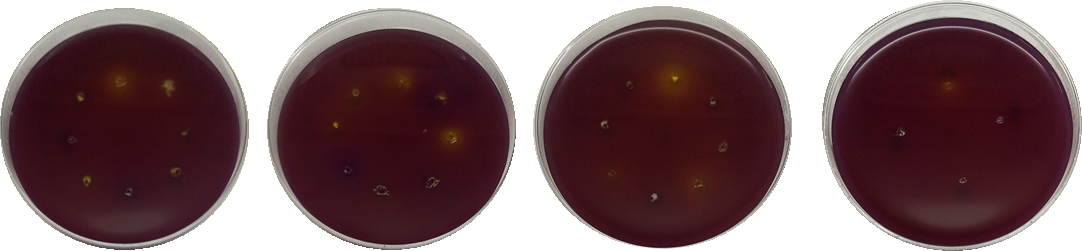

Supplement: Supplemental Information 5 — Evaluation of phosphate solubilization in isolates from soil samples from Tlalpujahua, Michoacán, Mexico, cultivated in Pikovskaya medium incubated at 30 °C. The yellow rings surrounding the colonies were measured daily. [file peerj-11-14697-s005.png]

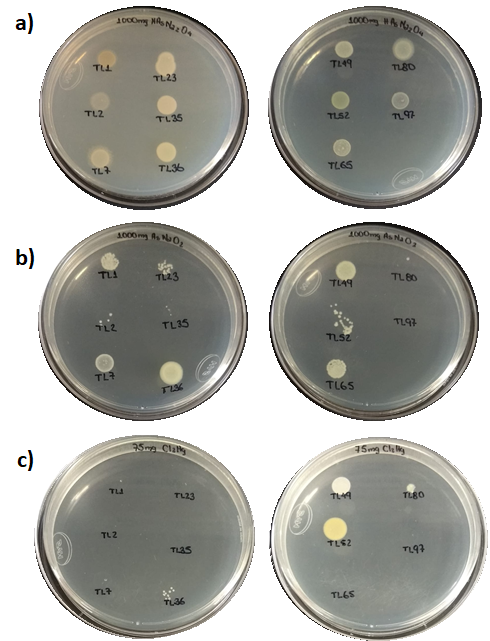

Supplement: Supplemental Information 6 — Evaluation of tolerance to heavy metals of isolates from soil samples from Tlalpujahua, Michoacán, Mexico. 1,000 mg/kg (meta) arsenite (AsNaO2) (a). 1,000 mg/kg sodium arsenate dibasic (HAsNa2O4) (b). 75 mg/kg mercuric chloride (Cl2Hg) (c). [file peerj-11-14697-s006.png]
